# Editorial for the Special Issue on Solid-State NMR Spectroscopy in Materials Chemistry

**DOI:** 10.3390/molecules25122720

**Published:** 2020-06-12

**Authors:** Mattias Edén

**Affiliations:** Physical Chemistry Division, Department of Materials and Environmental Chemistry, Stockholm University, SE-106 91 Stockholm, Sweden; mattias.eden@mmk.su.se

**Keywords:** structure, order, dynamics, density functional theory (DFT), magic-angle spinning, dipolar interactions, biomaterials, glasses, minerals, polymers

Nuclear Magnetic Resonance (NMR) has, over the past few decades, emerged as the most powerful spectroscopic technique for studying molecular structure across a sub-nanometer scale, as well as for probing molecular dynamics over widely spanning timescales (ns to s). Thanks to its applicability to matter in any physical state, NMR has become an indispensable characterization tool, used in almost any natural scientific discipline, including chemistry, physics, material science, earth sciences, biology, and medicine. In the latter area, the technique is known as magnetic resonance imaging (MRI), and offers a μm to m spatial resolution of the examined tissue. The pivotal importance of NMR/MRI techniques in modern science is mirrored in three Nobel Prize awards since 1991.

NMR exploits that the nuclei of many nuclides possess “spin”, which effectively makes them behave as microscopically small magnets. Owing to their magnetic properties, the nuclei interact with magnetic fields in their immediate surroundings. The sample to be examined is placed inside a super-conducting magnet and is subjected to intense pulses of radio-frequency (rf) irradiation, which results in an NMR spectrum. The position of each peak in the NMR spectrum reflects the *chemical shift* of a given (crystallographic) site, which depends on the local electronic (“chemical”) environment of the nucleus. More than 30 elements of the Periodic Table are well-suited for NMR studies. For NMR applications to organic substances and biologically relevant molecules, such as proteins and nucleic acids, the nuclides ^1^H, ^13^C and ^15^N are the most commonly exploited, whereas NMR investigations of inorganic and hybrid organic/inorganic materials additionally often target ^11^B, ^17^O, ^27^Al, ^29^Si, and ^31^P, as well as numerous other nuclides.

The present Special Issue concerns only NMR applications to solid phases (“solid-state NMR”). NMR on single crystals normally offers the most accurate NMR parameters. Zeman et al. [1] present such a single-crystal ^27^Al and ^14^N NMR study of aluminium nitride, from which valuable parameters relating to the electronic structure were extracted. Yet, such single-crystal NMR studies are nowadays rare: solid-state NMR experiments are merely conducted directly on powders of micrometer-sized particles under so-called *magic-angle spinning* (MAS) conditions, where the powder is spun at a tens-of-kHz rate. This procedure improves spectral resolution by suppressing NMR-signal broadenings from spin interactions, which are unproblematic in NMR spectra recorded either from single crystals or from the liquid/solution state, but which give very broad NMR responses from powders unless MAS is used. Solid-state NMR experimentation offers a nuclide-specific and non-invasive probing of molecular dynamics and/or the local structure, regardless of whether the nuclei are located in highly ordered crystals, amorphous solids (such as glasses), or heterogeneous composite/multi-phase materials. These features render (MAS) NMR an excellent complementary technique to those offering longer-range structural information, such as diffraction/scattering techniques and electron microscopy.

This Special Issue comprises five research articles and three reviews that together cover highly diverse aspects of solid-state NMR applications for probing structure or dynamics of materials. The review articles illustrate how one- and two-dimensional MAS NMR experimentation may be used for studying various polymeric systems, such as polyaniline [2], assemblies of borane-phosphane frustrated Lewis pairs [3], as well as borophosphate glasses [4]. Moreover, Lim and Kim [5] present an investigation of the thermal properties of the hybrid perovskite (CH_3_NH_3_)_2_CoBr_4_, along with a MAS NMR probing of the bearings on the ^1^H and ^13^C relaxation properties (NMR-signal damping) from the paramagnetic Co^2+^ centers; the results were contrasted with those reported earlier from an analogous complex involving the diamagnetic Cd^2+^ ion [5].

A progressively exploited area over the past two decades concerns the use of density functional theory (DFT) to calculate NMR parameters. Besides the fact that DFT-derived data greatly assist NMR-peak assignments, they are valuable for deepening the insight into which structural factors that primarily affect the NMR observables, as well as for rationalizing structure–property relationships. The “NMR crystallography” field derives from such a combination of experimental NMR and DFT computations to provide structure refinements, notably so for proton positions in polycrystalline materials, thereby offering a useful complementary tool to neutron diffraction. In this Special Issue, Sorte et al. [6] report a study of several Mg-based minerals by combining DFT calculations of ^1^H chemical shifts with results from advanced ^1^H NMR experimentation using dipolar interactions (see below). Moreover, Zeman et al. [1] present DFT calculations of ^27^Al and ^14^N NMR parameters of AlN.

The magnetic properties of the spin-bearing nuclei imply that the magnetic field generated by one nucleus is sensed by its neighboring nuclei: the *dipolar interaction* between two nuclear spins is mediated directly through space and depends on the inverse cube of their distance. This is extensively exploited to obtain specific and accurate information about (for instance) inter-atomic distances and geometries of molecular fragments. In this Special Issue, Zujovic et al. [2] and Knitsch et al. [3] review the utilization of such through-space dipolar-based MAS NMR techniques (along with their through-*bond* counterparts) to unveil the structure of polyaniline and borane-phosphane frustrated Lewis pairs, respectively.

The interatomic-distance-related information enabled by dipolar-based MAS NMR experimentation is particularly useful for structural characterizations of amorphous materials, where few (if any) other technique(s) than solid-state NMR may probe their medium-range (0.3–0.7 nm) structure. Here, Tricot et al. [4] review the utilization of such dipolar-based NMR techniques in the context of borophosphate glasses. Moreover, another research avenue of solid-state NMR concerns the development of new rf-pulse sequences for extracting structural information, such as NMR parameters that reflect the local structure, as well as distance/proximity information provided by the dipolar interactions. An article in this Special Issue by Yu et al. [7] presents new rf-pulse schemes for probing (for instance) the inter-connectivities of borate groups in B-bearing glasses via ^11^B–^11^B dipolar interactions. Yu et al. demonstrated their technique on a borosilicate glass, but it is equally applicable to the borophosphate counterparts reviewed in reference [4].

The majority of NMR studies in materials chemistry targets characterizations of new man-made materials, including biomimetic ones that seek to mimic Nature’s materials assembly and organization; one example concerns materials that approximate the complex multi-level structure of bone and tooth, which manifest a hierarchical organic/inorganic composite that involves fibrils of the protein collagen, along with an inorganic nano-crystalline calcium phosphate component (bone mineral). However, bone/tooth tissue may be studied directly by MAS NMR, as for instance exploited in the report by Tsai et al. [8], who contrasted the phosphate environments in dentin from rats and humans by using ^1^H and ^31^P NMR, including experimentation utilizing ^31^P–^31^P dipolar interactions. Dentin mineral exhibits a comparatively ordered calcium hydroxyapatite portion that co-exist with amorphous calcium phosphate. Tsai et al. [8] found that the proportions of phosphate groups in the “ordered” and “disordered” domains differ between human and rat dentin, where the latter comprises a significantly larger fraction of amorphous calcium phosphate relative to human dentin.

It is our hope that the set of articles compiled in this Special Issue gives the reader some flavor of what solid-state NMR may offer as a characterization tool of both natural and synthetic materials.
